# VARIATIONS IN FAMILY AND COMMUNITY RESOURCES AMONG DIVERSE AGING IMMIGRANT POPULATIONS

**DOI:** 10.1093/geroni/igae098.1564

**Published:** 2024-12-31

**Authors:** Fengyan Tang, Yanping Jiang, Lydia Li

**Affiliations:** Chair; Co-Chair; Discussant

## Abstract

Older immigrants rely significantly on family and community resources to address social isolation and related health issues. Yet, the formation and characteristics of these resources may vary within and across diverse aging contexts. Five studies in this symposium provide findings among Older Korean, Mexican, and Chinese Americans. Two studies present findings from the Study of Older Korean Americans (SOKA). Jang and colleagues examined the heterogeneity of family typology and its associations with psychological distress. Five family types were identified: close-knit, intimate but distant, detached, connected but dysfunctional, and dysfunctional. Park and colleagues identified five types of community: safe/integrated, safe/distant, moderate integration, marginal, and vulnerable. Findings showed that living in disadvantaged families and communities was associated with loneliness and mental distress. Using data from the Hispanic Established Epidemiologic Study of the Elderly Caregiver Supplement (H-EPESE CG), Rote examined the role of caregiver background, family and neighborhood support for depressive symptoms among Mexican American caregivers. Findings illustrated that Spanish-speaking caregivers showed a greater increase in mental distress, which was partially explained by lower caregiver resources. Two studies present findings from the Population Study of Chinese Elderly in Chicago (PINE). The objective and subjective aspects of social isolation had differential effects on mental and cognitive health, underscoring the importance of neighborhood resources and support system in buffering the negative health effects of social isolation. Collaboratively, these studies enhance our understanding of the variations in family and community roles across diverse aging contexts and suggest the needs for building resources to promote healthy aging.

